# Correction: Navigating groundlessness: An interview study on dealing with ontological shock and existential distress following psychedelic experiences

**DOI:** 10.1371/journal.pone.0338060

**Published:** 2025-12-03

**Authors:** The PLOS One Staff

## Notice of Republication

This article was republished on November 18, 2025, to correct errors in headings and in Fig 2 that were introduced during the typesetting process. The publisher apologizes for the errors. Please download this article again to view the correct version. The originally published, uncorrected article and the republished, corrected articles are provided here for reference.

## Supporting information

S1 File Originally published, uncorrected article. (PDF)

S2 File Republished, corrected article. (PDF)
