# Supplementary material for: Navigating groundlessness: An interview study on dealing with ontological shock and existential distress following psychedelic experiences
Source: PLoS One. 2025 May 5;20(5):e0322501. doi: 10.1371/journal.pone.0322501 (PMC12052184; doi:10.1371/journal.pone.0322501)
Supplement: Semi-structured Interview Guide. Table of all themes and subthemes with frequencies, percentages of sample and indicative quotes — (DOCX) [file pone.0322501.s001.docx]

**Supplementary Materials**

for Argyri et al., 2025

*Navigating Groundlessness: An interview study on dealing with ontological shock and existential distress following psychedelic experiences*

**Semi-structured Interview Guide**

**Guidance to interviewee: “This interview will be focusing on one particular psychedelic experience and the subsequent experiences after it. This should be the same experience you wrote about in the questionnaire**.”

*If the participant is unclear which experience they referred to in questionnaire/hasn’t seen the reminder email, remind them by reading out their difficulties verbatim from the survey.*

1. **Circumstances of the experience**

- How old were you at the time of the experience in question?
- How many years ago was this?
- What substance(s) did you take - and what dose (if known)?
- Did you have previous experiences with psychedelics?
- Tell me about what was going on in your life in the months prior to the psychedelic experience?
- What was your intention for the psychedelic experience? And did you have any expectations?

1. **The experience itself**

Could you tell me more about the psychedelic experience that you described in the questionnaire *Probe for context of experience, including others involved and interactions, duration of experience, emotions*

1. **Extended difficulties**

**Guidance to interviewee: “I’d like to discuss the difficulties you experienced *after the effects of the psychedelic substance had worn off.* In the online questionnaire, you mentioned…”** *(include extract written that emphasises existential struggle element)*

Guidance for interviewer: Focus on the effect of experiences and subsequent events *on beliefs, sense of meaning, emotional impact*

1. **Management of difficulties / coping**

- Tell me about what coping strategies you employed, or any help that you sought from others in dealing with and integrating these difficulties?
- And what did you find *un*helpful?

1. **Insights on integration, change and support**

- Tell me about any changes you experienced in relation to your belief system at the time, and since then?
- What does integration of a psychedelic experience mean *to you*? Do you feel that you integrated this experience over time? If so, in what way? If not, in what way was it not integrated?

1. **Suggestions for supporting others in the future**

- What sort of support or services would you like to see to help people going through similar experiences?

**Table of all themes and subthemes with frequencies, percentages of sample and indicative quotes**

| **Themes and subthemes** | **Frq** | **% sample** | **Indicative Quotes** |
| --- | --- | --- | --- |
| **Life Contexts** |  |  |  |
| Highly transitional time | 12 | 46% | *It was not only that I feel like this was like sandwiched between these trips, but I was also sandwiched in a very transitional place in my life... (Caitlin)* |
| Turbulent or stressful time | 12 | 46% | *My life was a screaming wreck at that time. I was coming off of less than a year after him being gang raped on a ski trip. I was in a family home with an alcoholic abusive stepfather. I mean, you want to line up the shit things going on in my life. At that time, it was pretty intense! I was a junior or senior in high school. All my friends were college students, and significant drug users. (Cal)* |
| Period of healing and self-exploration | 9 | 35% | *So, the pandemic sort of brought me to a point of like, absolute rock bottom. But that was a good thing, because it dropped, I finally started properly healing myself after a lifetime of trying to but failing. And it was with psychedelics, it was just me and myself, no therapist, no coach, and mentor, nothing, just me and a book, which is complex PTSD, from surviving to thriving by Pete Walker, and a bottle of LSD, essentially. (Adrienne)* |
| No significant personal difficulties | 2 | 8% | *It was quite a relaxed time where I had enough free time to be able to explore such things... (Will)* |
| Socially isolated | 2 | 8% | *I was living in a city where I moved two years prior or three years prior for work, and I didn't have any friends in the city or family. My closest friends and family were like an hour and a half away. I had been pretty isolated. (Kirsty)* |
| **Intentions** |  |  |  |
| Exploration or curiosity | 9 | 35% | *I'm a psychonaut I'm interested in exploring the mind and spiritually curious. I'm curious what maybe, you know, I'm open to seeing what this has to offer either way (Caitlin)* |
| Having fun | 7 | 27% | *So I don't think I had a specific intention, beyond just having a good experience! Only fun with friends and exploring this combination of LSD and MDMA. (Noah)* |
| Active search for healing | 6 | 23% | *And I just started doing like a 100-microgram [LSD] trip every week and applying Pete Walker's [author of Surviving to Thriving] re-parenting method, and then discovered that there was more than one inner child, I went down the internal family systems road and was just like, right, I'm going to save all my inner children. And I've just spent about eight, nine months doing that (Adrienne)* |
| Positive prior experiences | 5 | 19% | *I previously experienced very, very positive states on LSD. But I was struggling to work out like how to repeat those experiences. So I was just interested in just to keep exploring. Just because those have been the best experiences of my life so far, so it was good to keep going in that direction and see what happened. (Will)* |
| Self-development and spiritual Growth | 5 | 19% | *I think it spiritually and psychologically matured me very fast in a way that I imagined could have happened over you know, years or decades even. (Caitlin)* |
| Clarity on life decisions | 5 | 19% | *So I was thinking about moving back to where my family and friends were, which is in the Philadelphia area of Pennsylvania, and thinking about a new job. So I was hoping that maybe a trip would give me some clarity (Kirsty)* |
| Inspired by psychedelic research and media hype | 3 | 12% | *I've become really interested in the psychedelic research, particularly the Johns Hopkins stuff. So I basically set up my room as though it was one of these, like [session rooms]. But I guess the only thing that was missing was someone to watch over me. (Steve)* |
| Seeking a spiritual experience | 2 | 8% | *I got interested in spirituality, a lot of Eastern stuff. So I've always been quite like, philosophically minded. I've listened to people like Terence McKenna. I come from a small town. It's very mundane. And I'm listening to this guy on the internet, talk about these mind-blowing experiences. And I will just like, I've actually written in my diary, I was like, ‘give me the keys to the universe’. The reality that's presenting itself is so mundane. I felt like, there’s gotta be more and now it's just like a search. Like, I just want to be blasted out, you know, I just wanted to like, see what it was all about. (Steve)* |
| **Worldview Shifts** |  |  |  |
| Shift away from materialism | 8 | 31% | *I think before, when I studied neuroscience, I knew conceptually of the problem of not being able to explain consciousness, you know, our materialistic worldview. What this did to me was integrate the spiritual or– the integrating the spiritual sounds like I'm no hippie who believes in crystals–, no, like, like no, but I am more open or less focused on this purely scientific materialistic worldview somehow. This experience, just kind of cracked it open (Fred)* |
| Loss of faith in their previous idea of God | 4 | 15% | *My relationship with spirituality absolutely changed because at that time in my life, I was on the edge. I was considering become a rabbi and I had become pagan. And I was on this very spiritual cognitive dissonance. I think the fact that no other power came down to help me in this huge time of need may have been part of the shift. If I want change to happen, I have to do it. Which of course shifted me away from going to be a rabbi and [towards] becoming a priestess. (Cal)* |
| Undermining their faith in psychedelics | 4 | 15% | *But going from a position where I felt that I could trust this substance almost, or that it would always work out well for me when I did this substance. It had been a guiding light. And then suddenly, something had changed. (Harry)* |
| Spiritual to scientific worldview | 2 | 8% | *I've written a lot about natural science. And I'm just basically trying to reconstruct a worldview that's in line with reality. I'm trying to try to stay as close to what we actually know as possible, rather than deal with these kinds of things that are all the way over there. (Steve)* |
| Now agnostic | 2 | 8% | *I don't have any kind of religious model as such... I do obviously believe that there is something after this life and, you know, but I don't hold on to It's this or it's that. I don't know. Yeah. So, again, it's, was that a literal thing? Or was it more of a metaphorical thing? I don't know. So I just hold it loosely (Georgia)* |
| **The acute psychedelic experience** |  |  |  |
| **Existential concern** | **16** | **62%** |  |
| Perceived physical or ego death | 9 | 35% | *So I just went “fuck it, I'm gonna die”. And I just let go. And I just, it was almost like allowing someone to execute me, you know, just like put a sword right through my chest. (Theo)* |
| Overwhelming responsibility | 7 | 27% | *But what I can say legitimately is, I think it changed, inherently changed, me. I still don't think the world is all that real. I really think we create our universe to an enormous extent. We create it, God isn't making this happen. Somebody else isn't making this happen. The buck stops right here. And self-responsibility is huge. (Cal)* |
| Solipsistic isolation or Aloneness | 6 | 23% | *I was having this really deep experience with my guide, that we were truly one person. And I was like, Oh my God, that's so depressing. Right? Like, like, is there no novelty in the world? Right? Like if this is all in my head, like, Oh, what a sad small world, right?... to realize that it's all actually happening in my head was the most depressing thing I could think of...If this is all in my head, it’s just a dream - it's meaningless. (Aaron)* |
| Meaninglessness or emptiness | 5 | 19% | *It led to this mental, this place where life was totally meaningless. It was all facets of that... it was like, 'Is my life meaningless? Or, erm, empty? (Aaron)* |
| **A feeling of No Exit** | **14** | **54%** |  |
| Fear the experience will never end | 8 | 31% | *As I experienced it was this empty aloneness that I desperately wanted to escape life and existence started to feel like a prison that I can't get out of. It was as though I was stuck in some kind of solipsistic, psychedelic nightmare version of Groundhog's Day, that I was profoundly alone and that this nightmare called life on loop eternally and that there was nothing I can do about it. I had a distinct experience of absolutely no agency. I had no control. My sense of emptiness, meaninglessness and imprisonment became overwhelming. (Max)* |
| Fear of insanity and permanent damage | 6 | 23% | *Like I have done something like deeply irreparable to my existence. This sense of like, oh my god, like I'm never going to be the same. My body feels different. I feel different in my body (Caitlin)* |
| Fear of Dying | 2 | 8% | *It was over... like I don't want it to be. Like I'm not ready for it. You know? Like what about my god-daughter’s christening? ... then I was sort of– I wanted I guess to try to negotiate that I will come back to real life. Because I wanted to have children. I wasn't ready to - like I felt really strongly that that wasn't the way that it was meant to be (Clara)* |
| **Confusing uncertainty** | **12** | **46%** |  |
| Inability to compute, or articulate the experience | 8 | 31% | *One of the recurring feelings was like, 'Okay, so we're all one. AKA I am God, like, truly I am God, I'm infinite. I am the universe.' That's awful, and impossible to process. (Aaron)* |
| Received confusing message | 6 | 23% | *It was like it was like being at the cosmic circus. I forgot what it was that I was trying to remember. And the closer I got to remembering it, it was like these the good side of the trip would come out and then I forgot it and it would just be chaos again. It was like losing my mind, and the main theme of it was these alien sort of entities that were communicating to me in this alien language. I've forgotten what it was to be a human. I kept saying, human and kept muttering ‘psilocybin’. Like it was this word, and I was trying to remember, what is that thing? (Steve)* |
| Questioned sexual orientation | 2 | 8% | *It kind of frightened me I think it awakened some kind of like dormant homophobia I had and then that was it was like scary and unpleasant. I started to have thoughts like that if I want to accomplish my dreams and I want to get where I have to get in life then I have to realize that I'm homosexual and stuff like that. It went for a while and felt very real and very confusing.... This kind of existential thing... felt like really a message from beyond, a message from the universe that you'd have to give up women, you have to give up your sexuality if you want to be whatever.... (Noah)* |
| **Ontologically Challenging Experiences** | **17** | **65%** |  |
| Challenging mystical or religious experience | 8 | 31% | *I was occupying something like unity consciousness. I was sometimes referring to myself in the third person. I no longer had a connection to a sense of self – the word no longer carried any reality whatsoever. I did not experience this loss of sense of self as a positive thing at all… (Max)* |
| Given insights or gnosis | 7 | 27% | *It was kind of a Gnosis moment of understanding things I hadn't understood and kind of seeing the source (Beth)* |
| Hellish afterlife | 6 | 26% | *Just hell, like the worst images that I could possibly imagine. And while I was experiencing these images, I just thought how the hell does my brain come up with images like this?... And there was also such a sense of evil, and it was just like the worst kind of Satanist people that you could possibly imagine were in this dream or were in this journey. And there was blood, and they were just awful symbols. (Georgia)* |
| Perception of an entity encounter or possession | 6 | 23% | *I was trying to remember what it felt like to be human. I became as they were, I no longer knew who I was, who my family was, I became this alien and was communicating in this strange, ugly language that helped me gain this super intelligence that allowed me to understand the universe in ways that isn't conceivable. (Steve)* |
| Extra-Sensory Perception & Out-of-Body Experiences | 4 | 15% | *We were seeing kind of at the same time, all these strange buildings and things. And it was really it was disturbing. Like it was really disturbing stuff, what we both saw. And then so I felt that they had left like slightly and I said to him, like they were really in a rush to do that. And I knew we knew we were saying the same thing. You know, he was describing it to me, he felt the same and he was talking to... I could just see him talking to them. He could see me. (Beth)* |
| Time travel and Transformations | 2 | 8% | *As far as I was concerned, in that moment, I had gone back 1000 years, there was no sense like “you're in a psychedelic drug, you're visualising and seeing something, eventually this will pass and you will return to it” - it was gone. And I was sat around....I was sat in a cave with ancestors, and we were looking at the fire just talking, we all kind of dressed in kind of like ragged clothes. (Theo)* |
| Expressions of fear | **16** | **62%** |  |
| Anxiety or terror | 11 | 42% | *The magnitude of the fear and the panic that I was experiencing was unlike anything that I've ever encountered. (Max)* |
| Paranoia | 7 | 27% | *I was also slightly convinced that like the CIA would come because I thought this, you know, this information is too too esoteric for me to have access to (Beth)* |
| Feeing unsafe | 4 | 15% | *It amplified my internal compromised safety. Extremely amplified it and I felt extremely unsafe. I couldn't trust, I I literally felt like I literally felt like I was on a thread. Like I was holding on to my dear life on a thread. (Emine)* |
| **Other aspects of the acute phenomenology** | **14** | **54%** |  |
| Overwhelm | 8 | 31% | *It was just so overwhelming. Like like full on sensory overload to the max I think yeah, no, like memory didn't work anymore (Fred)* |
| Aggressive reaction | 2 | 8% | *I tried to physically fight with my guide, tried to punch him. Actually, I did punch him. I didn't try. I wrecked his house. Not like lasting damage, but like I was in his kitchen, and he had a fruit bowl and I threw it into the ceiling. Trying to provoke something that felt like it wasn't ‘coming from within me’ via violent acts (Aaron)* |
| **Exiting the trip** |  |  |  |
| Shaken, fragile and vulnerable | 9 | 35% | *Coming out of the journey just sobbing crying, despondent soul-tearing despair about this, emptiness. ... it was brutal. I was coming out of this in the fetal position on a bed crying and sobbing. (Aaron)* |
| Anxious and overwhelmed | 6 | 23% | *It was just like sensory overload everything just felt really overwhelming (Kirsty)* |
| Physically Exhausted | 2 | 8% | *I felt as if I had definitely, physically as if I had been in a battle. So physically my body was finished. (Georgia)* |
| **Extended Difficulties** |  |  |  |
| **Ontological shock and existential confusion** | **26** | **100%** |  |
| Experiences of derealization and questioning reality | 15 | 58% | *It felt like I was in a golden state of derealization. Yeah, dissociation but also sometimes very associated, like being very much in the moment, not being able to think about what's going to happen the next minute... It's hard to put into words... It felt as if I was a ghost somehow... I was there but also I was not there. (Ida)* |
| Obsessive preoccupation with making sense of the experience | 12 | 46% | *I could not stop thinking about what I had seen...I was just like, couldn't focus on anything. It was just going round and round in my head like that.(Beth)* |
| Continuous confusion and struggle to accommodate ontological shock | 11 | 42% | *The psychedelic experience was so powerful that it fundamentally challenged my perception of reality, and how well I could trust my perceptions in the day-to-day... the thing over the long term was ontological. I just wasn't 100% sure whether... I could never really — and to this day that's true — really trust my sense of what was actually going on (Elijah)* |
| Existential crisis and despair | 10 | 38% | *Most days I would feel anxiety, fear, sit in disorientation and deeply saddened by my existence, which progressed into existential crisis… the questioning of this whole universe, why are we here, what's the point of this. (Emine)* |
| Emptiness, meaninglessness and nihilism | 8 | 31% | *There was a lot of nihilism as well. Those extreme levels of annihilation of not wanting to do anything. What's the point of showering now? We're all gonna die anyway. (Emine)* |
| Questioning identity | 8 | 31% | *My sense of self, I mean, it was gone, you know. It was like it had been obliterated into a million pieces. And I couldn't really work out who I was. I just had this, like, routine of going to work. And I just stuck to that as a means of like, keeping some sense of inner structure by having some outer structure. And, like, if I'm honest, if I just, if I'd sat and thought about that for a while, for the first few weeks, I think I would have just cried because like, it didn't seem very clear who I was anymore. (Theo)* |
| Grief over the loss of their past self | 8 | 31% | *I would really love to somehow get back to how I used to be... [Back to] when I didn’t used to think and have all this knowledge. That sort of ignorance is bliss, sort of mindset... there’s a sense of loss for like the person I was because yeah, it’s just gone. (Elijah)* |
| Religious or spiritual disappointment and betrayal | 7 | 27% | *I went looking for God and for love and for connection and I got the exact opposite. gave me this feeling of existential betrayal that to this day, I feel has created a very, I mean, profound distrust for me, of a very deep sense of like not feeling safe in life. (Max)* |
| Psychotic and paranoid experiences | 7 | 27% | *There were voices, many voices in my head that were psychotic, thoughts that were just so ridiculous. So twisted, so dark, so, so weird, disgusting, fearful, all the crazy mix of strange thoughts over the simplest of things (Jan)* |
| Fear of insanity or permanent damage | 6 | 23% | *I had this deep sense inside of me over those three months that I lost all my skills and not going to be able to work anymore. 'You're stupid now, you're a drug-induced insane person.' (Youssef)* |
| Feeling of being visited or possessed | 6 | 23% | *And I would perceive it as negative energy that I was absorbing and could not access my breath anymore to try to clear my nervous system, clear my energy body... eventually there were audio hallucinations. And so I felt as though they were out to get me for some reason, like these entities from others were out to get me. (Cora)* |
| Preoccupation with mortality and death | 5 | 19% | *For the first time I started thinking of death. So my mortality, I mean, we all know death exists, but for the first time, it was in my bones in my mind every second (Will)* |
| Spiritual grandiosity | 2 | 8% | *I had some grandiosity around it like 'I am the shit basically', like I can maintain the state of bliss, bliss, like reality is just giving me everything I want. ...I was living in like, a God Realm. Everything was flowing beautifully in my life...I had kind of entangled my identity with that as well as being like, spiritual superior. But...then I started losing stability. (Cora)* |
| **Persisting emotional difficulties** | **17** | **65%** |  |
| Fear and anxiety (including physical) | 12 | 46% | *I felt like, like I can't breathe. Like I don't have any air to breathe… very extreme restlessness. Like I have to do something with myself and if I don't…I'm going to die. (Noah)* |
| Hopelessness and suicidality | 5 | 19% | *I experienced my own death, which was uncomfortable but also peaceful. The problem is this has led to being suicidal and feeling that nothing actually matters and maybe I’d be better off ending this life. (Teri)* |
| Fear of being alone | 4 | 15% | *I had real difficulty with staying alone. The anxiety was overpowering. (Noah); Like somehow being on my own suddenly felt more scary or anxious. (Kirsty)* |
| **Social difficulties** | **16** | **62%** |  |
| Social Isolation | 14 | 54% | *It kind of just took me into this very inward hibernation. I just didn't want to be around people or, you know, talk to anyone and just worked through that. (Jessie)* |
| Difficulty with social interactions | 6 | 23% | *I had the feeling that all my scales were open, like everything could just enter…. I was absorbing everything from everyone around me... I was being very anxious about my relationships. (Ida)* |
| **Functional disruptions** | **12** | **46%** |  |
| Severe disruption in ability to work and study | 11 | 42% | *I quit the job. Because it was too…I, I couldn't continue... I didn't have any structure at all. It was super stressful. But yeah, thereafter I just completely collapsed. (Ida)* |
| Attention issues and disorientation | 4 | 15% | *I couldn't focus. I was a very avid reader, I could read 100 pages a day easily and would want to read more and stop. After that ceremony, I couldn't get through five pages without physically being in pain and fidgeting and wanting to leave. (Youssef)* |
| **Somatic Problems** | **10** | **38%** |  |
| Sleep issues | 7 | 27% | *Sleeping was returning to hell. (Aaron) I kept on waking up and just going over and over with the, with the images and with everything that came up. (Georgia)* |
| Bodily discomfort | 4 | 15% | *I was so uncomfortable in my skin. I was crawling out of my skin. I never felt more uncomfortable in my body and like, scared in this way of like, my body doesn't feel like mine (Caitlin)* |
| **Trauma and PTSD similarities** |  |  |  |
| Traumatic experience | 12 | 46% | *It was really traumatizing on me and my nervous system (Cora)* |
| Flashbacks and other forms of re-experiencing | 15 | 58% | *For the first six months after that… like maybe every three to four weeks flashes of terror... I was having a visual effect of the world starting to dissolve and it was like a flashback - a recurrence of the journey (Aaron)* |
| **Triggers back to experience** | **15** | **58%** |  |
| Sleep related | 8 | 31% | *The act of falling asleep felt too much like the act of going into the journey experience again. That, especially like that microsecond moment as your body's noticing you're falling asleep. It's like, [impression of fearfully catching breath], and I come back awake. So that was awful. (Aaron)* |
| Meditation and breathwork | 7 | 27% | *Don't meditate. Meditation is the worst because you'll just go more in there. Just distract yourself” (Fred)* |
| Substance use (including further psychedelic trips) | 5 | 19% | *Anytime I would smoke cannabis in fairly large amounts, I would get triggered: sometimes falling into the same headspace that I was the night of that first experience. If the setting was off, it would be very very tough for me. (Youssef)* |
| Discussions related to psychedelics or the content of their trip | 4 | 15% | *I remember going for a walk with a friend, a hike. And I don't know, just somehow the conversation turned to this place [that triggered re-experiencing] (Kirsty)* |
| **What helped manage the difficulties?** |  |  |  |
| **Grounding practices** | **22** | **85%** |  |
| Meditation and mantras | 10 | 38% | *I think with the meditation, it's really important to find a sort of balance [with] turning inwards and doing introspection, and I was too caught up into that. And then I was not experiencing the world outside of me. Because I was so close in my thoughts, I didn't understand anything. So I kept closed in my head, I got stuck in my head. But then, from that point, on, I started to go outward again. Trying to experience like what am I feeling? What am I hearing? What do I see? And just objectively naming it, trying to not like judge things, just try to observe. And, yeah, these are both meditative practices, but...one is going inward, the other one is going outward, and I really needed to go outwards. (Ida)* |
| Time with animals and nature | 8 | 31% | *I'd go for walks by myself in this lovely forest there, that was really helpful actually going for like quiet, solitary walks in the forest. Just like feeling that sense of spaciousness and the sense of calmness of the forest. You know, there's a bit of inner chaos in your mind, like a forest can be like a really soothing balm to that. (Theo)* |
| Yoga and other body work | 7 | 27% | *Some kind of bodywork, some kind of work or practice or therapeutic approach which works with the body and not just with the mind with the thought, because I feel that if I didn't have the tools to focus my attention on what goes on in the body, I think it would have been a lot more difficult. (Noah)* |
| Creative expression | 6 | 23% | *I picked up the guitar. After a year and so from playing the guitar, I can tell you that this practice saved my life… It… puts all the worries, all of the panic, all of the stress away… The world just drops. It's just me and the guitar, me singing. My heart soars, my mind quiets down. I feel grounded. (Youssef)* |
| Experiences with water | 5 | 19% | *Having a bath was both grounding and relaxing (Will) There are forces of nature that are far greater than me, that can tolerate without any difficulty, my inner turmoil, like it didn't scare the wave away, it didn't change the course of the waves trajectory. That way, it was like this, I was smashed by nothing... the cold water immersion, just that sensitive state change. And then when you are surfing the flow state, there's this pure- there's this like, beautiful sense of excitement of just like, the wave is constantly adjusting, and you're constantly adjusting to it. And there's, there's no time to stop and analyse it, or think about what you're doing (Theo)* |
| Physical exercise | 4 | 15% | *Spin cycling [was] super intense, like you're spinning on the spinning bikes with techno at the same time. An American instructor up front was like just like shouting at you. I loved it so much because it means you can't think of anything else! (Fred)* |
| Attention-focus practices | 4 | 15% | *One of the things that I did was a simple countdown practice. So…when I was getting into obsessive thinking or high anxiety regarding the experience, I would count down from three…So not fighting against the desire to anxiously think or ruminate…but clearly labelling myself, this is here, but this isn't helping. Then I would redirect my attention back to my body into the present moment and just try to be with my life as it was happening in the moment. (Max)* |
| Trauma release exercises | 4 | 15% | *I felt as if I was kind of outside my body so so just doing things like just stretching, yoga, and then a TRE, which is I think it's trauma release. That's where you get your body into a state and your legs shake and that's, they use it for, yeah, releasing trauma... it was like There's trauma in my body and I've got to get rid of it.... I've got to shake, I've got to get this stuff out of my body. And then things like just walking barefoot. So you know, again, luckily, I know about grounding [laughs]. So, yeah, I just felt like I just needed to have my feet on the grass and that sort of thing (Georgia)* |
| **Cognitive practices** | **17** | **65%** |  |
| Cognitive distancing | 9 | 35% | *It's a distancing thing. I think the version that I have since come to is, 'What is this part of me trying to tell this other part of myself? What can I learn from this?' And I find that particular framing really helpful because it both creates that mental distance to analyze and notice what's happening. (Aaron)* |
| Acceptance and letting go of need for answers | 9 | 35% | *The mind’s always trying to grasp onto something but… the mind will never get there. It’s coming to terms with infinite complexity rather than trying to figure it out...Now it's just about really making peace with the fact that maybe I won’t know, and it's just about really stopping myself from trying to figure things out. I was treating it as a philosophical exploration sort of thing. When really, it's quite like a visceral experience. (Steve)* |
| Shifting perspective from objective reality to inner state | 5 | 19% | *What I saw on my trip was just that – the quality of my mind and how it was determining the quality of my life. The trip showed me in what direction my mind was pointed.. that helped me strip this sense of objectiveness from my experience, and allowed me to connect with the sense that what I experienced was not true objectively. It was simply true and relevant to me. And that's something that definitely helped stabilise me a lot… (Max)* |
| Journaling | 4 | 15% | *Journaling as well to just write the whole journey out so I could go back and I could read it… wrote probably about three or four pages, and then I would go back and just read one paragraph, and then just work on that. And then maybe the next day, go and do another. So it was all down there and then I could just deal with little bits every day as I was sort of integrating, rather than having everything here that was just this whole kind of mess of stuff (Georgia)* |
| Distracting activities | 4 | 15% | *Watching Friends for distraction [helped] (Fred) Listening to game of thrones audiobooks to distract myself [helped] (Elijah)* |
| Self-compassion and gratitude | 3 | 12% | *Gratitude...just makes you a happier, better human being. But also it helped me reframe a lot of these things? Like, okay, I don't understand existence, but I'm alive. Isn't that great? Like, look at life out there. It's amazing… let's just be grateful...to be able to live... to not have died, and to [make] the most of it (Fred)* |
| **Self-education and prior experience** | **15** | **58%** |  |
| Frameworks for understanding | 10 | 38% | *I read about spiritual emergency and realised you know, there is a way out of this. You think like that's it - I've really done it this time, there's no going back again, like I'm gonna be crazy for ever. So hearing that narrative that that wasn't the case was just lifesaving itself. I understood the significance of what I was going through as something that could be overcome (Beth)* |
| Learning about others’ challenging experiences | 8 | 31% | *Another more minor one, which helped alleviate a lot of the attachment to the experience was actually reading and listening to the experiences of others. (Max)* |
| Prior life experiences | 5 | 19% | *Because of my age, I have done a lot of work... having more life experience and just being interested, perhaps, in things like consciousness and psychology and, so just well read on all of those subjects. And then just life in general, when you get to 56, 57, you've dealt with quite a lot of trauma anyway! (Georgia)* |
| **Interpersonal support** | **22** | **85%** |  |
| Reaching out to and finding community | 13 | 50% | *I discovered a spiritual awakening sharing circle...that was literally my only support because I was going through this all alone, living alone in my flat. But every month I had this group I would go to, and for five minutes I would talk about this crazy stuff I was going through with this demon and people didn't judge me. And that was what kept me going (Adrienne)* |
| Talking to friends and understanding others about experience | 11 | 42% | *What helped definitely was talking about it. And knowing that I do have very sacred women in my life that I could tell this experience to that wouldn't judge me that would hold space for me and, you know, offer their unconditional love and compassion (Jessie)* |
| Talking to others with psychedelic experiences | 7 | 27% | *Just having someone say that it's just like a normal thing that happens and like just kind of I remember it just being really reassuring and just knowing that it was okay, that someone else had been there and had some nice advice. (Will)* |
| Feeling understood: seen, heard, believed | 6 | 23% | *Talking with friends, I think that helped because I felt seen and heard and held. (Jessie)* |
| Being with friends /and family | 2 | 8% | *If I was on my own it was the feeling of wanting to be near someone. Like just having a friend nearby or family nearby like comforting just added a layer of security for me. (Kirsty)* |
| **Behavioural and practical help** | **16** | **62%** |  |
| Therapy and medication | 8 | 31% | *I started therapy, and was also working with my primary care doctor on medication. And so I think the combination of therapy and starting medication also really helped (Kirsty)* |
| Returning to prior normality, familiar people, habits | 5 | 19% | *Even if I felt completely nihilistic about what I was doing. I just made myself just go eat the food, eat sleep, get the exercise. Because I knew that was gonna help. So I tried my best just to sort of keep to habits and not let myself go down the rabbit hole (Theo)* |
| Positive life decisions and life changes | 4 | 15% | *And so once I kind of made one decision, I feel like now it's like a domino effect that I'm like, Okay, now let's make the next decision. And let's make the next one. After that, and slowly but surely, I can, like shape my life again in a different way. So I think that was like an affirming way to be like to get myself unstuck, just like finding one decision or one change to make and making it (Teri)* |
| Flexibility and understanding at work | 2 | 8% | *I let my boss at work know that I was really struggling. So I felt grateful I had like that support as well in the workplace. (Kirsty)* |
| **Spiritual Practices** | **11** | **42%** |  |
| Further psychedelic 'journeys' | 6 | 23% | *I saw... really evil, horrible, disgusting images again… But what I was dealing with there, came out it was fear. And so once again, I kind of almost had to go to hell. And then through that Ayahuasca journey, I was able to release so much fear. (Georgia).* |
| Non-psychedelic spiritual practices | 5 | 19% | *I then went on to have an out of body experience for about 30 seconds during that retreat… it showed me that there was plenty of challenge that can be can be undertaken without ingesting a consciousness altering drug (Theo)* |
| **Perceived unmet needs and suggestions for the future** |  |  |  |
| **Increased awareness and societal infrastructures** | **18** | **69%** |  |
| Structures for sharing and support during and after | 9 | 35% | *Even at festivals there's tents like PsyCare. Most festivals usually have a place where people having hardships can go, which is great... knowing that there's friendly people who are just there to help, usually in a comfy setting where you can just go and hang out or ask them help or to just be there like, it's just really reassuring. Also online versions of that could exist (Beth)* |
| Access to information on support structures | 8 | 31% | *I think knowing maybe about therapists who were more specialised in like integration support would be helpful (Kirsty)* |
| Public awareness of potential harms of psychedelics | 8 | 31% | *People need to know that psychedelics does not mean it's a quick fix. I think it's so important that you know what you're getting into. Because it's not always going to be universal love and the flowers talking to you, sometimes it's going to be going to hell (Georgia)* |
| Legalization and decriminalisation | 5 | 19% | *Just not feeling like you had to be afraid of, like, who you talk to about it, like am I gonna get in trouble if I talk to the wrong person about this? So I think the legal– I actually think, like, legalising the drugs will help tremendously (Teri).* |
| Awareness there is a way through the difficulties | 3 | 12% | *Knowing that there is a path through and... also keep trying, don't give up. That there is something else on the other side (Kirsty).* |
| **Hindsight: advice to others** | **13** | **50%** |  |
| Journeying with informed others | 6 | 23% | *And in hindsight, had I had somebody with who was like, if the sober tripsitter was somebody who was very experienced in psychedelic territory that I could have told like, I'm having– I'm having this really disturbing experience right now. That very well could have been the connection, the interaction that I needed in that moment, to work through whatever fear was being brought to the surface rather than being completely captured by it. (Max)* |
| Develop a toolkit of resources | 5 | 19% | *To have a basket of different things that you can use… So that people would know that they have those resources, if you have a challenging psychedelic journey, these are the resources that you can try. (Georgia)* |
| Dedicate time for integration after the experience | 3 | 12% | *I wish I even just committed to one hour every week of writing my journal about that experience, and trying to process it (Teri)* |
| Start small, go slow | 3 | 12% | *In hindsight I feel like maybe having done a smaller dose before might have been helpful (Kirsty)* |
| Come into experience with respect and caution | 2 | 8% | *It's just about respect. It's about knowing that what you're doing is, you know, you, you come to it with a certain amount of caution, respect, reverence. You realize that what you are going to, it's altering your mind, it's altering your consciousness, it's taking you to places that you can't go to without the substance. So I think, coming to where you light a candle, and you pick flowers from the garden, and, you know, we always smudge ourselves too, well we say Well we don't know what we're really doing but we, you know. And we have special food ready for afterwards and all that kind of stuff. It just creates a container where you're coming into to do work, in just a respectful way and I think yeah, for me that is, that's important.* |
| **Concerns about psychedelics** | **12** | **46%** |  |
| Underestimating harms | 8 | 31% | *I think we have vastly underestimated the cost. And if you're going in with open eyes, sure. But I came back literally crying and racked and months of literal hell. Hell. Like, my definition of hell is the experience I had. There's benefits out of it. Right? But you got to go in open-eyed. (Aaron)* |
| Issues within psychedelic associated cultures | 4 | 15% | *I realized the dysfunction in the Ayahuasca and New Age spirituality hype. And there's a lot of spiritual bypassing going around in those circles, and how that's a bit contagious. Which is why I cut off ties with a lot of people from that circle, but also maintained ties with people who came to similar realizations. And people who actually do these things less as a status symbol, less as a symbol that, 'Hey, I'm better than other people’ more ‘I'm just a human trying to be human better.' (Youssef)* |
| Risks of co-dependency and abuse | 3 | 12% | *My wish… is that we help people to become more autonomous rather than codependent even months after their sessions. And how do you do that? By continually reinforcing the self-agency and helping them see and become more aware of their own challenges in the mundane. (Emine)* |
| Commercialisation | 2 | 8% | *Why has ayahuasca become so popular or so mainstream?...I really do believe all the answers are inside of us and we can connect with that just by meditating. But it's almost become like this product that we're selling, like, you know, try this shampoo. It's gonna make your hair look great. (Jessie)* |
| **What was unhelpful in dealing with extended difficulties** |  |  |  |
| **Lack of support and understanding from others** | **18** | **69%** |  |
| Lack of community and others to speak to | 10 | 38% | *The fact that I didn't have anyone I could speak to... not having a community before I did that was probably very unhelpful (Georgia)* |
| Others’ spiritual interpretations of their experience | 8 | 31% | *What's so scary is that he would pin everything to an entity possession, which was even scarier. So, instead of taking ownership of this compromised facilitation, instead of taking responsibility, he would say oh, you must have been possessed by entity. (Emine)* |
| Lack of broader cultural understanding of psychedelic experiences | 4 | 15% | *You do this and you have this amazing experience and unlock these connections within, like amongst people in nature and in the world, and yet you're stuck in a life where not everybody understands that and it's very isolating (Teri)* |
| Lacking integration support from their guide | 3 | 12% | *They were very absent. I remember emailing them once and just being like, I don't know what's happening. Can you help or anything you could advise? and they were like ‘Do more medicine’... Not helpful. (Emine)* |
| Not getting professional help | 2 | 8% | *I didn't speak to a therapist for 3 months out of fear of being sectioned (Youssef)* |
| **Escapist and obsessive coping** | **14** | **54%** |  |
| Further substance use | 9 | 35% | *I took a high dose again. And though, psychologically, I came back down much faster, and felt like me myself, the same loop came up. (Harry)* |
| Meditation | 3 | 12% | *This meditation was some sort of way to flee from myself. I was continuously busy with getting away from myself because I was so not accepting myself because I had the feeling that something was wrong with me. (Ida)* |
| Obsessive search for intellectual answers | 3 | 12% | *Researching existential matters made things worse…trying to intellectually solve the experience, trying to think my way out of it…to obsessively find a book or a talk or something. Some magical sentence about the nature of self and reality that is going to cure my anxiety… The search for the magic bullet, that is definitely not an effective strategy. (Max)* |
| **Understandings of integration** |  |  |  |
| A continuous process (never complete) | 8 | 31% | *I think it's gradual, and it's an ongoing process. I think I'm still accepting. And so you can see there's evidence and I'm still processing it, right? So, no, it's not a lightbulb. It's it's a journey. (Aaron)* |
| A learning process of adopting new worldviews | 8 | 31% | *In our darkness, when we're not resisting it, and not running away from it, there are deep, deep lessons and teachings inside the darkest depths of our experiences...at the bottom of the deepest, darkest oceans of experience, we can find the most profound, most life changing wisdom. The Darkness has so much wisdom, if we can just stay in our hearts and be open to what the lesson is that life is giving to us… I don't even believe in so called 'bad' anymore. Yes, I will never harm another person. But I don't believe there is 'bad' in a sense, because all the bad things we see have a beautiful lesson. They're pushing us to be better people, they're pushing us to do better, to help our fellow people. So if the so called Bad things are just inspirations to be better and to do good in the world, then how's that bad thing actually bad? It's doing good. It's going good in a greater sense, it's teaching us... There is a way out. And it's possible. I'm the proof that it's possible (Jan)* |
| A bridge between the metaphysical and ordinary | 4 | 15% | *Going from the mystical reality and the ordinary reality and yet bridging the gap, letting them co-exist. You know, I don't have to not have conversations about car insurance… like learning to deal with that side of things, the yoga of going from the mystical to the mundane and learning to bridge that gap and the disparity between them and not feel like you're betraying either one side... I can live in both those sides at once now. I can go to work and I can go and have a crazy trip and neither really imposes on the other. (Beth)* |
| Successfully letting go of the experience | 2 | 8% | *It means just coming into peace and acceptance of what the experience was. And I want to say understanding, but it's more acceptance. Because I'm not sure it's all understandable. So, acceptance is more realistic. And I think that I feel it means to feel more embodied. So, in those areas of my body where I've got this sort of feeling of aversion to those experiences, like feeling more in those parts of my body, where I'm trying to shut out a feeling. And just being more lighthearted, easygoing, joyful, you know, generally with further integration, it's like, you're letting– putting a heavy weight down (Theo)* |
| Answering of questions brought up | 1 | 4% | *I think integration to me is as if I came out of the experience with unanswered questions to have them answered in some sort of way, even if doesn't have to be like a classic, rational answer, but just a sense of I had this question answered, and also kind of owning up to parts of me which I maybe rejected in the experience, which made it difficult. So in that sense, I feel like it's pretty integrated. (Noah)* |
| **Positive outcomes and learnings** |  |  |  |
| **Self-development and spiritual growth** | **14** | **54%** |  |
| Psychological maturation | 7 | 27% | *I think it spiritually and psychologically matured me very fast in a way that I imagined could have happened over you know, years or decades even. (Caitlin)* |
| Expanded awareness | 5 | 19% | *I became a lot more aware of my thoughts, more mindful (Beth)* |
| Helped make positive life decisions | 5 | 19% | *I made decisions after that point that were just like yeah, it was just more clear what felt aligned and worth it (Caitlin)* |
| Felt empowered through overcoming challenging experiences | 4 | 15% | *I feel massively resourced, that if I can handle that and get through it then I can kind of handle anything. (Clara)* |
| New sense of purpose | 2 | 8% | *It's a paradox because I was stressed and scared. But as it went on, and as I protected myself. I went down the rabbit hole of all of this and it was like a massive purpose. (Beth)* |
| Humbled by their experience | 2 | 8% | *It's a huge part of my life story... I've grown to know the importance of experiencing the full range of human emotion. And just like really accepting the fullness of being human, so I feel more human... I've been humbled in a way... being human. I'm able to see and integrate the complexity of what it is to be human and that feels really meaningful, personally, and in my work (Cora)* |
| **Improved mental health** | **3** | **12%** |  |
| Recovery and cessation of prior treatments | 3 |  | *[I stopped setraline] because I just didn't feel the need for it anymore because I have been taking life way too seriously. And now, I didn't really need to because it's not that serious. And the Gnosis experience and seeing God, realising that I have much more choice and power over my mind. And these decisions are my emotions. I just, yeah, I just felt more autonomy in my being and that just I didn't need it...some of the depression was answered by some of the things I saw during the Gnosis, you know, seeing God seeing my past lives and seeing that the universe, there's much more to it. That just that massively helped with me not taking everything so serious and getting so angry getting so depressed. It was comical. (Beth)* |
| **Increased prosociality** | **12** | **46%** |  |
| New sense of compassion, love, understanding | 8 | 31% | *It's given me compassion, so for example, with this awful thing that's going on in Israel, I've been actually, when I've been meditating and praying for peace there, I've been able to actually go into sort of like those terrorists and think Oh how must they be feeling? You know, they've committed all these horrible, atrocious things, but what trauma are they living with?... And so I've been able to almost go into like their evil acts and hold it with compassion. And I think, you know, largely that's got to do with the fact that I went there, I went to hell. And so in a way, working with that, you know, although it's, I mean, I would rather not have those images in my mind, but I've tried to use them in like my practice, my compassion practices (Georgia)* |
| Inspired to help others | 7 | 27% | *That inspired me to delve into modalities and training with an intention to help educate others and support their journeys. (Emine)* |
| Realising value of connection with others | 4 | 15% | *It made me value again, the friendships or the family or the relationships that I have, you know, more strongly, more gratitude for that. (Jessie)* |
