# Supplementary material for: Navigating groundlessness: An interview study on dealing with ontological shock and existential distress following psychedelic experiences
Source: PLoS One. 2025 May 5;20(5):e0322501. doi: 10.1371/journal.pone.0322501 (PMC12052184; doi:10.1371/journal.pone.0322501)
Supplement: Attachment — Submitted filename: Comments.docx [file pone.0322501.s002.docx]

**Overall Evaluation:** The topic of the article is important and suitable for publication.

**Introduction:**

1) Please provide more detailed information on the types of psychedelic substances. Clarify the inclusion of cannabis (in the table for Cora & Don) and MDMA (for Ida), and explain why these are considered psychedelics.
2) Please expand on the ‘renaissance’ in psychedelic research.
3) While the research emphasizes the potential shortcomings and harms of psychedelic substances, the introduction should also briefly address the benefits that have contributed to the resurgence of interest in psychedelic research in recent years.

**Method:**

1) Include the reflexive aspect and researcher characteristics in the methodology, particularly in relation to their connection with psychedelic substances.

**Table 1:**

1) Age at Interview: For Aaron, the age range is listed as 45-55. If this is an estimation, please indicate that it is an assessment.
2) Inconsistencies in Age- Please correct these inconsistencies: Georgia is listed as 55 at the interview but 56 at the psychedelic experience. Similarly, Jessie is listed as 35 at the interview but 36 at the psychedelic experience. Chris is listed as 27 at the interview but 34 at the psychedelic experience.
3) For Youssef and Cora: The age ranges for their interviews and psychedelic experiences are unclear. Please clarify.
4) What substance is "Rape" (for Jessie)? Please provide clarification.
5) In the “Substance/s taken” column, does this indicate that the participants experienced difficulties after taking the listed psychedelic substances? For Aaron, you mention "different trips"—did he encounter difficulties during all of them? This needs clarification. And for Beth, is the reference to polysubstance use in one event? Similar clarification is needed for Cal and Clara.

**Figures 1 and 2:**

1) Figures 1 and 2 are somewhat blurry and difficult to read; please address this issue.
2) Titles should be added to the figures. Within the article text, include placeholders like “[Figure 1 insert here]” and “[Figure 2 insert here]” where appropriate.
