# Supplementary material for: Navigating groundlessness: An interview study on dealing with ontological shock and existential distress following psychedelic experiences
Source: PLoS One. 2025 May 5;20(5):e0322501. doi: 10.1371/journal.pone.0322501 (PMC12052184; doi:10.1371/journal.pone.0322501)
Supplement: Attachment — Submitted filename: Response to Reviewers.docx [file pone.0322501.s003.docx]

**Response to Reviewers for Manuscript PONE-D-24-20547**

**Title**: *Learning from Groundlessness: An interview study on dealing with ontological shock and existential distress following psychedelic experiences*

**To**: Dr. Michal Mahat-Shamir, Ph.D., Academic Editor

**From**: Eirini Ketzitzidou Argyri
 **Date**: November 10th, 2024

Dear Dr. Mahat-Shamir,

Thank you for the opportunity to revise and resubmit our manuscript. We appreciate the detailed and thoughtful feedback from the reviewers, and we believe their comments have significantly helped improve the quality of our paper.

Below, we have provided a point-by-point response to each of the reviewers’ comments, outlining the corresponding changes made to the manuscript. Please also note we have made a minor edit to the title to match the theoretical framework we added as part of the requested revisions.

First to address your points for additional information and clarification:

- PLOS ONE's style requirements: We have made amendments accordingly
- Funding: As requested we are copying the amended Funding statement here, including the role of the funders:

“JE received funding from Emergence Benefactors, the William G. Nash Foundation and the Sarlo Family. EKA also received funding from the Economic and Social Research Council UK. The funders had no role in study design, data collection and analysis, decision to publish, or preparation of the manuscript. "

- Data sharing restrictions:

The ethical approval from the University of Greenwich Ethics Committee (researchethics@greenwich.ac.uk) precludes sharing of the data. The full interview transcripts, although anonymised, contain potentially identifying information. Available data relevant to this study is presented in the manuscript text and in Supporting information.

- Captions for Supporting Information files: We have added those at the end of the manuscript

**---------------------------------------------**

**Responses to reviewers' comments:**

Reviewer #1 points:
General

- Proficient literature framework discussing the effects of psychedelics on the human brain lacking, especially information processes

We thank the reviewer for the opportunity to revise and expand our theoretical framework. We make reference to existing theoretical framings for the action of psychedelic substances, specifically where they relate to belief changes, including the reviewers recommendations. However we also note we are not neuroscientists and this is a phenomenological paper, as such brain processing theories are beyond the focus of the paper and we don’t expand on them. We did take the opportunity to dive deeper into the literature that is fundamental to the existential focus of the paper and we are grateful to the reviewer for their suggestions.

Specifically we have strengthened, throughout the manuscript, both our discussion of existential literature, the potential psychological mechanisms where they relate to beliefs and help explain existential distress, framings of trauma (see below), and crucially our proposition of the concept of integration as transformative learning that follows pivotal mental states and the encounter with core existential concerns (ontological insecurity).

- More consideration on framing of trauma

Thank you for the pointers. Indeed many of our participants described their experiences as traumatic and their difficulties could be conceptualised within the clusters of ptsd criteria. We had originally considered this framework and moved away due to agreeing that what binds the findings instead is an existential focus and challenges with meaning making that relate to the ungrounding of prior beliefs and facing one's existential vulnerability. However, we have now expanded our discussion of trauma literature where relevant in both the introduction and discussion, acknowledging that trauma is amongst the potential outcomes and such framing, where it resonates with individuals, may also have therapeutic value in the management of associated distress.

- More literature on existential framing if this is the focus

See above, we have made considerable revisions to expand on this throughout the paper. Thank you for the invitation to do so.

Intro

- 1 1 What is meant by “use”

Clarification added.

- 1 1 physicalism , non-physicalist beliefs – should give a context, definition, example.

Definitions added.

- 1 2 Use a word other than trigger which may have a negative connotation.

Replaced with catalyse.

- 2 2 Integration- define it in this context, consider Dan Siegel’s work

We have now expanded our discussion on the concept of integration and make note of Dan Siegels work beyond psychedelics, thank you for the pointer. We feel our revisions and expanded discussion on the concept of integration has improved the paper.

- 7 “Different nationalities, relate to this?”
  We are not fully clear on what the reviewer is requesting. However given our sample size we are not in a position to compare our findings based on nationality. We do make the suggestion in the discussion for future studies to explore the role of cultural background. The table is provided for descriptive demographic info of our participants, not for analytical focus.
- 10 3 When describing experiences, add percentages not just numbers

Thank you for this feedback. In qualitative analysis, we aim to explore the depth and complexity of participants’ experiences rather than quantifying responses. While participant counts for each theme are provided to offer transparency, percentages could imply representativeness that may not align with qualitative goals (see Braun & Clarke, 2006). Furthermore, we have included percentages in the supplementary materials themes table, where they can be referenced as needed without impacting the interpretative focus of the main text.

- 10+ Was there a theme of bodily reactions/sensations? Described in exiting, does it have a space in itself even though these are commonly stated as a type of effect. The body is missing in this article.

The body is missing in the article as the focus (including of the interview questions) was on meaning making. Indeed many participants struggled with feeling disconnected from their body, stuck in their heads, hence the need for grounding. We have added discussion of this in the Discussion, including relevance to dissociation, trauma and relevant existential interpretation.

- 22 last Self education is actually part of cognitive practices, mentioned before perhaps branch them together and differentiate between the two types (based on external information)

We appreciate you noticing the ambiguity on this part of the results, we have resolved this.

- 30 core existential concerns (death, meaninglessness, responsibility, and aloneness)= seem more like traumatic symptoms related to cluster 4- as I said above.

As above, we take your point and have returned to this in the discussion, thank you.

- 28 “ Self development and spiritual growth “ can be related to the concept of posttraumatic growth .

Yes, thank you. We make further reference to interpretations of posttraumatic growth and link to the framing of integration as transformative learning that we propose.

- Using trauma-focused therapies, like EMDR to process the psychedelic experience as a traumatic memory, may help resolve PTSD-like symptoms during the integration period [71]. I agree. Discussion was built better, but following a conceptual framework for the lit review, I would reorganize.

Thank you for noting this, we have reorganised and expanded the Discussion, as above.

Reviewer #2:

Introduction

- 1) Please provide more detailed information on the types of psychedelic substances. Clarify the inclusion of cannabis (in the table for Cora & Don) and MDMA (for Ida), and explain why these are considered psychedelics.

Thank you for pointing this, clarification added.

- 2) Please expand on the ‘renaissance’ in psychedelic research.

We take your point, we have rephrased to resurgence of research and defined it in time.

- 3) While the research emphasizes the potential shortcomings and harms of psychedelic substances, the introduction should also briefly address the benefits that have contributed to the resurgence of interest in psychedelic research in recent years.

This has now been addressed in edits to the opening paragraphs.

Method:

- 1) Include the reflexive aspect and researcher characteristics in the methodology, particularly in relation to their connection with psychedelic substances.

Thank you for the suggestion, we have now added a section for reflexivity in the Method to clarify our positioning and process.

Table 1:

- 1) Age at Interview: For Aaron, the age range is listed as 45-55. If this is an estimation, please indicate that it is an assessment.

We have added additional clarification.

- 2) Inconsistencies in Age- Please correct these inconsistencies: Georgia is listed as 55 at the interview but 56 at the psychedelic experience. Similarly, Jessie is listed as 35 at the interview but 36 at the psychedelic experience. Chris is listed as 27 at the interview but 34 at the psychedelic experience.

Thank you for spotting these inconsistencies, they have been corrected.

- 3) For Youssef and Cora: The age ranges for their interviews and psychedelic experiences are unclear. Please clarify.

Clarified as above.

- 4) What substance is "Rape" (for Jessie)? Please provide clarification.

Clarified as above.

- 5) In the “Substance/s taken” column, does this indicate that the participants experienced difficulties after taking the listed psychedelic substances? For Aaron, you mention "different trips"—did he encounter difficulties during all of them? This needs clarification. And for Beth, is the reference to polysubstance use in one event? Similar clarification is needed for Cal and Clara.

Thank you for catching this ambiguity. Clarified as above.

Figures 1 and 2:

- 1) Figures 1 and 2 are somewhat blurry and difficult to read; please address this issue.

Addressed.

- 2) Titles should be added to the figures. Within the article text, include placeholders like “[Figure 1 insert here]” and “[Figure 2 insert here]” where appropriate.

We have added these.

------------------------------------

Thank you for considering this revised version of our work. We are confident that the improvements we have made in response to the reviewers' insightful suggestions contribute to a more robust and impactful manuscript. We look forward to your feedback and your decision regarding publication of our study in *PLOS One*.

Sincerely,
Eirini K. Argyri
University of Exeter
